# Optimizing biomagnetic sensor performance through *in silico* diagnostics: A novel approach with BEST (Biomagnetism Evaluation via Simulated Testing)

**DOI:** 10.1016/j.isci.2024.110167

**Published:** 2024-06-04

**Authors:** Chenxi Sun, Yike Liang, Xiao Yang, Biying Zhao, Pengju Zhang, Sirui Liu, Dongyi Yang, Teng Wu, Jianwei Zhang, Hong Guo

**Affiliations:** 1 State Key Laboratory of Advanced Optical Communication Systems and Networks, School of Electronics, and Center for Quantum Information Technology, Peking University, Beijing 100871, China; 2 School of Life Sciences, Peking University, Beijing 100871, China; 3 Faculty of Engineering, University of Bristol, Bristol BS8 1TR, UK; 4 School of Physics, Peking University, Beijing 100871, China

**Keywords:** biological sciences, computer science, physics

## Abstract

Advancing biomagnetic measurement capabilities requires a nuanced understanding of sensor performance beyond traditional metrics. This study introduces Biomagnetism Evaluation via Simulated Testing (BEST), a novel methodology combining a current dipole model simulating cardiac biomagnetic fields with a convolutional neural network. Our investigation reveals that optimal sensor array performance is achieved when sensors are in close proximity to the magnetic source, with a shorter effective domain. Contrary to common assumptions, the bottom edge length of the sensor has a negligible impact on array performance. BEST provides a versatile framework for exploring the influence of diverse technical indicators on biomagnetic sensor performance, offering valuable insights for sensor development and selection.

## Highlights

• Introduced the Biomagnetism Evaluation via Simulated Testing (BEST) methodology
• Found sensor proximity to source and short effective domain optimize performance
• BEST provides a versatile framework for evaluating biomagnetic sensor performance
• Future research can adapt BEST to more complex scenarios and classifiers

## Graphical abstract

**Figure:**
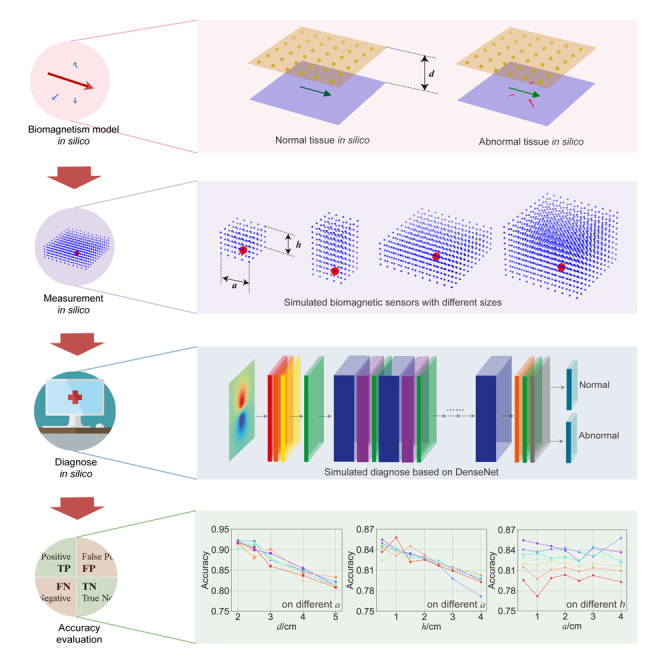

## Introduction

Biomagnetic signals are generated by bioelectrical activities among heart, brain, etc.^1^^,^^2^ Since the first measurement of biomagnetic signals,^3^ kinds of sensors have been developed, such as search coil, fluxgate, magnetoresistance, superconducting quantum interference device (SQUID), and atomic magnetometers.^4^ Mooney et al. designed a portable magnetocardiography (MCG) device with search coil magnetometers and proved that the device is capable of detecting MCG signals with both shielded and unshielded environments.^5^ Janosek et al. developed the 1-pT noise fluxgate magnetometer and used it to detect MCG signals in unshielded environments.^6^ Different kinds of SQUID magnetometers have been applied to the measurement of MCG,^7^^,^^8^ magnetoencephalography (MEG),^9^^,^^10^ magnetogastrography (MGG),^11^^,^^12^ etc. under different conditions. A comparison of the performance of measurement systems is also underway, for example, Liao et al. compared the first-order, four-vector, and second-order high-Tc SQUID gradiometers for MCG systems in unshielded and shielded environments.^13^ Recent advances in optically pumped magnetometer (OPM) technology have allowed OPMs to be possible substitutes for SQUIDs. In several successive studies, OPMs have been demonstrated capable for sensing biomagnetic signals generated by human’s heart,^14^ spontaneous alpha rhythm in human’s brain,^15^ and slow wave in rabbit’s gastrointestinal tract^16^ in unshielded Earth’s field. Compared with SQUIDs, lower system complexity of OPMs make more flexible placement of sensors around the body possible. Eichler et al. claimed that they studied the impact of the restricted spatial coverage and sensor density on slow wave tracing performance *in silico* and discovered that larger coverage above the segmental venter of the torso and higher sensor density have positive effect on the tracing performance.^17^

In spite of the sensitivity and the distance to the skin, the biomagnetic sensors vary in the sizes of the sensing domain, which is the space area for the magnetometer sensing the magnetic field. For instance, we can approximately regard it as the cylinder within the coil for the SQUID, the atomic cell for the OPM, and the magnetic core for the fluxgate magnetometer. To distinguish the size of the device, we define the size of the sensing domain of the magnetic field as the effective size. The effective size could influence the detection of abnormal signals. Sensors with large sensing domains may encounter many nearby dipoles. Stronger magnetic fields from high-current dipoles can overshadow weaker ones, creating a "masking effect" on measurements. Additionally, more dipoles can lead to frequent cancellations of their magnetic fields, minimizing the group’s overall impact—an "offsetting effect." Both effects can hinder the detection of small tissue lesions. However, there are few previous studies focusing on the performance of biomagnetic sensors influenced by the effective size.

Experimentation *in silico* refers to the simulation of biological experiments on computer systems, rather than in a laboratory setting. It allows modeling biomagnetic fields in a controlled environment. Numerous computational models for biomagnetic fields have been established, attempting to recreate and study the magnetic fields produced by biological currents. These models include modeling bioelectric volume sources, such as the current within the heart, and volume conductors, such as the human body. When considering the volume source, the current dipole model has been demonstrated to be efficacious in describing the physiology of electrically active biological tissues.^18^^,^^19^ The current dipole model was initially devised to study bioelectric signals. In a seminal work, W. Einthoven, G. Fahr, and A. de Waart made use of a fixed-location dipole of variable orientation and magnitude as the volume source of the electrocardiogram.^20^ Nowadays, researchers have simulated the bioelectromagnetic fields generated by the brain,^21^ heart,^22^^,^^23^ and gastrointestinal tract^24^^,^^25^ using current dipoles as volume sources. The position, orientation, and magnitude of dipoles in these models vary over time. Dipoles are determined either phenomenally in reference to the results of physiological experiments^21^^,^^22^^,^^24^ or biophysically through an electrophysiological model of organs.^23^^,^^25^ In addition to bioelectromagnetic fields generated by healthy organs, pathological signals caused by distinct diseases, including gastroparesis^26^ and coronary heart disease,^27^ have been simulated through simple modifications of the general model.

The application of machine learning in the analysis of bioelectromagnetic signals has made significant advancements, with some of the studies demonstrating substantial practical value. For example, Angelaki-Kaxiras et al. combined a random forest model with single-lead electrocardiogram (ECG) for hypertension detection, achieving an diagnose accuracy rate of 81%.^28^ In the field of machine learning, convolutional neural networks (CNNs) have provided good results in object classification tasks. It has been used to extract different kinds of features from biomagnetic signals. Based on CNN, Tao et al. proposed the MCG-Net for fine-grained delineation and diagnostic classification of Q-, R-, S-, and T-waves from MCG data.^29^ Apart from biomagnetic studies, techniques developed in bioelectric signal analysis can be easily transferred to biomagnetic signals due to certain similarities between the bioelectric and biomagnetic signals. Rajendra et al. applied CNN for automated detection of myocardial infarction using ECG data and obtained an average accuracy of diagnosis up to 95.22%.^30^

The goal of this study is to develop a framework to evaluate the influence of effect size on the performance of biomagnetic sensors. In this study, we use current dipoles to simulate the biomagnetic fields generated by a healthy heart or a heart with lesions. The biomagnetic fields are detected by sensors with different effective sizes computationally, and the results of each type of sensors are randomly divided into training set and validation set. The training set is used to optimize the CNN classification model, in order to classify the results by whether lesions are in their source models or not. While testing with validation set, the classification accuracy is used as the criterion to evaluate the performance of the corresponding type of sensors. We name this framework Biomagnetism Evaluation via Simulated Testing (BEST). Experiments under BEST conclude that for a sensor array, the closer the array is to the magnetic source and the shorter the sensors in the array, the better the performance of the sensor array, given the number of channels in the sensor array, the bottom area of the sensors in the array will not significantly affect the performance of the array.

## Results

### Model establishment

We built models to simulate magnetic signals generated by a normal heart and a heart with lesions as the subject of simulated detection by sensor arrays in BEST. The models were built in a virtual three-dimensional (3D) space. For the convenience of the subsequent description, we established a coordinate for this three-dimensional space. In this coordinate, we defined plane y=0cm and plane y=24cm as the left and right sides of the trunk, respectively, and plane z=−16cm and plane z=16cm as the abdominal and back sides of the trunk, respectively, and the above four planes together with plane x=0cm and plane x=24cm constitute the thoracic cavity. In this primary model, the above definition of the position of the thoracic cavity was an attempt to simulate the human body. At the same time, the model also ignored the effect of the volume conductor, which was attributed to the negligible influence of the volume conductor on the z-direction magnetic field that we were about to measure.^31^^,^^32^

Based on the definition of the position of the thoracic cavity in the models, we adopted the current dipole model to simulate biomagnetic field generated by a normal or defective heart. The magnetic field of a normal heart was generated by a dipole located at (12cm,12cm,0cm), with an orientation (1,0,0), and an intensity of 10 units. In order to simulate the biomagnetic field generated by a defective heart, we randomly added dipoles to the normal heart model to represent the lesions. Specifically, in order to be compatible with the size of the real heart, the x-coordinate of the added dipoles was randomly selected within interval [10cm,14cm], y-coordinate within interval [10cm,14cm], and z-coordinate within interval [−1cm,1cm]. The orientation of the dipoles was randomly selected, and the current intensity of the dipoles was randomly selected between 0 and 1 unit. The number of dipoles that met the abovementioned conditions was a random integer in the range of 1–10 (Figure S1). Such a combination of dipoles jointly simulated a heart with lesions. In order to consider various possible lesions as comprehensively as possible and make the experimental results more universal, we generated 10,000 sets of dipole combinations.

**Figure 1:**
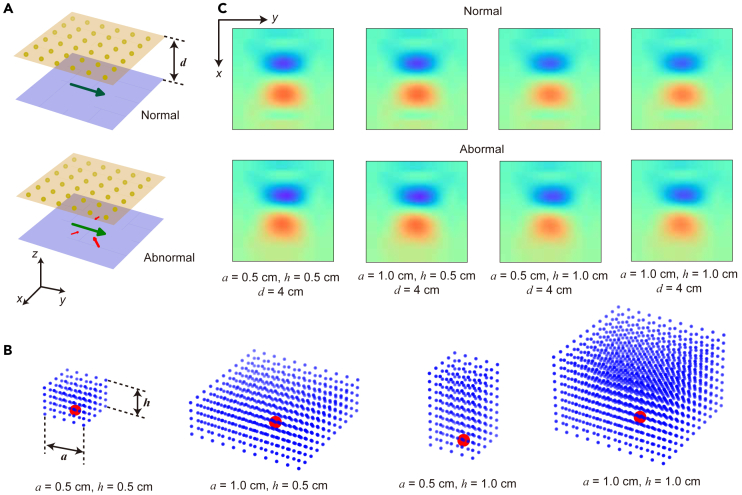
Model establishment and data acquisition (A) Model of MCG signals and placement of sensors. Green and red arrows represent current dipoles simulating MCG signals. The midpoint of the arrow handle represents the position of the dipole, the direction of the arrow represents the orientation of the dipole, and the length of the arrow represents the strength of the dipole. The largest dipole is fixed in position to simulate the normal MCG signal (above), whereas the other dipoles are randomly generated to simulate lesioned signals (below). The orange dots represent the position of the center of the sensors’ bottoms. The blue plane and the orange plane represent the planes where the largest dipole and the sensors’ bottoms are located, respectively. The distance between the two planes is defined as d. (B) Models of cubic and cuboid sensors. The red dot represents the center position of the sensor’s bottom. The blue dots represent all field points that need to be included in the average calculation when simulating the measurement result of the sensor. The bottom edge length of the sensor is defined as a, and the height of the sensor is defined as h. (The diagram shows the cases where a=0.5cm,h=0.5cm, a=1cm,h=0.5cm, a=0.5cm,h=1cm, and a=1cm,h=1cm, respectively). (C) The simulation results. Raw measurement results from 36 sensors were superimposed with random noise and then interpolated using two-dimensional (2D) cubic interpolation to obtain a magnetic field distribution map. The color represents the magnitude of the magnetic field. (The diagram shows the simulated measurement results of a=0.5cm,h=0.5cm, a=1cm,h=0.5cm, a=0.5cm,h=1cm, and a=1cm,h=1cm sensors, respectively. The top three figures show the simulated measurement results of a normal heart model, whereas the bottom three figures show the simulated measurement results of a heart model with simulated abnormalities).

Figure 1A provides an example of the abovementioned model, where the subfigure above is the model simulating magnetic field generated by a normal heart, the subfigure below is a model simulating magnetic field generated by a defective heart, and the dipoles simulating lesions in the right subfigure are one of the 10,000 dipole sets mentioned earlier.

### Data acquisition

To simulate the measurement results of the abovementioned models at different distances with sensors of different effective areas, we established a virtual sensor array consisting of 6×6 sensors. To simulate the sensor array, we identified a plane parallel to the ventral surface of the torso in the anterior direction and defined the perpendicular distance between this plane and the biomagnetic source (such as the cardiac muscle) as the parameter distance (d). Next, we uniformly selected 6×6 points on this plane to represent the positions of the centers of the sensors’ bottom surfaces. The distance between adjacent points was set to 4cm.

We used cuboids with different bottom edge lengths and heights to simulate the effective domains of the sensors, with the bottom edge length defined as parameter a and the height defined as parameter h. The centers of the bottom of these cuboids coincided with the centers of the sensors’ bottoms (Figure 1B).

In our study, the distance between the bottom of the sensors and the source was set at d=2cm, 2.5cm, 3cm, 4cm, and 5cm, respectively. The bottom edge length of the sensors was set at a=0.5cm, 1cm, 1.5cm, 2cm, 2.5cm, 3cm, and 4cm, respectively. The height of the cuboid sensors was set at h=0.5cm, 1cm, 1.5cm, 2cm, 2.5cm, 3cm, and 4cm. The design intention of these parameters was to simulate different biomagnetic sensors measuring the MCG signals in actual experiments.

After setting the positions of the cuboids representing the effective domains of the sensors, we selected a field point every 1mm within the effective area, and the z-direction magnetic field at each field point is calculated separately (Figure 1B). We took the average value of the magnetic field at each point in a certain cuboid as the measurement result of the corresponding sensor. In this way, we roughly simulated the measurement results of a sensor with z-directional magnetic field sensitivity.

For a sensor array with a specific effective domain at a certain distance, one normal measurement result was obtained on the model simulating the normal heart’s biomagnetic signal, and 10,000 abnormal measurement results were obtained on the 10,000 models simulating the defective heart biomagnetic signal corresponding to the previously mentioned 10,000 dipole sets, respectively.

The above measurements did not take the noise in actual measurements into account. To simulate the noise, we generated 10,000 6×6 matrices composed of 36 random numbers following a normal distribution. For a sensor array with a specific effective domain at a particular distance, each random matrix was added to the previously obtained normal measurement results and a certain abnormal measurement result, thus giving rise to a one-to-one correspondence between 10,000 abnormal measurement results and 10,000 random matrices. In this way, 10,000 normal final measurement results and 10,000 abnormal final measurement results were obtained.

For all 6 × 6 final result matrices, we expanded the final result matrices to 128×128 matrices through spline interpolation. In Figure 1C, we visualized the measurement results with noise obtained by different sensor arrays.

### Size and distance effects

We selected the first 8,000 matrices from the set of matrices with normal measurement results and the first 8,000 matrices from the set of matrices with abnormal measurement results, as the training set to train the model. The remaining 4,000 matrices were used as the validation set. We performed the model training on the High-Performance Computing Platform and evaluated the quality of the data by taking the classification accuracy of the best-performing model on the validation set (Figure S3A), which was obtained after 30 epochs of training (Figure S3B), as the evaluation of the sensor array’s performance.

**Figure 2:**
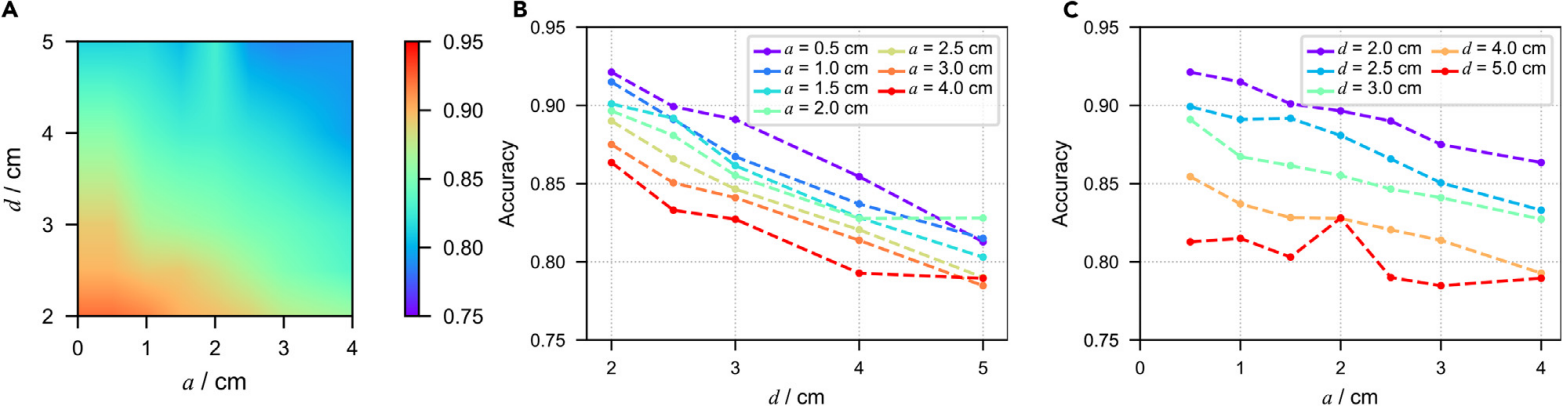
Size and distance effects for cubic sensor arrays (A) Linear interpolated result of accuracy on a-d parameter space; a represents the bottom edge length, d represents the distance from the main dipole plane, and the color represents the accuracy of classification on the validation set. (B) Scatterplot of accuracy variation with the change of d under the condition of fixing different values of a. (C) Scatterplot of accuracy variation with the change of a under the condition of fixing different values of d.

**Figure 3:**
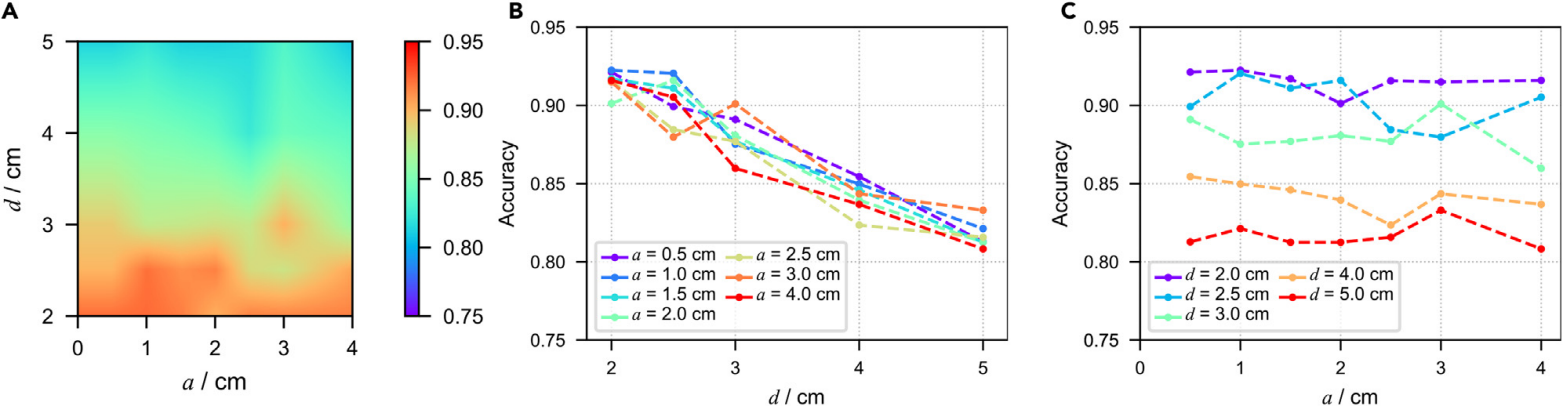
Size and distance effects for cuboid sensor arrays with h=0.5cm (A) Linear interpolated result of accuracy on a-d parameter space; a represents the bottom edge length, d represents the distance from the main dipole plane, and the color represents the accuracy of classification on the validation set. (B) Scatterplot of accuracy variation with the change of d under the condition of fixing different values of a. (C) Scatterplot of accuracy variation with the change of a under the condition of fixing different values of d.

**Figure 4:**
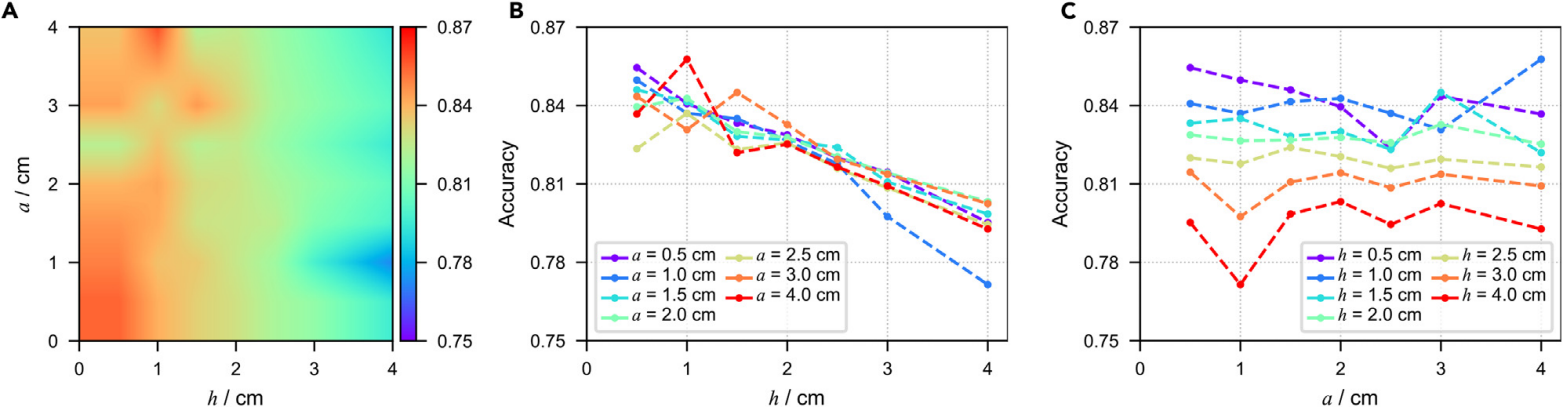
Size effects from height and bottom edge length for cuboid sensor arrays with d=4cm (A) Linear interpolated result of accuracy on h-a parameter space; h represents the height of the sensors, a represents the bottom edge length, and the color represents the accuracy of classification on the validation set. (B) Scatterplot of accuracy variation with the change of a under the condition of fixing different values of h. (C) Scatterplot of accuracy variation with the change of h under the condition of fixing different values of a.

We reorganized the data from the simulated cubic and cuboid sensors, respectively. With the bottom edge length of the effective domain as the x axis and the distance from the sensor’s bottom to the main dipole plane as the y axis, we used colors to represent the corresponding accuracy of the sensor array on a two-dimensional (2D) parameter space. The blank area between the data points obtained from the simulation experiment was filled by 2D linear interpolation (Figures 2A, 3A, and 4A).

The results show that for sensor arrays composed of sensor with the same effective domain, the further the distance between the sensor array and the main dipole plane, the lower the classification accuracy, which holds for sensors with different effective domains. The relationship between accuracy and distance is more intuitively demonstrated in the graph that depicts the relationship between accuracy and distance while fixing the shape and size of the effective domain (Figures 2B and 3B). This may be because biomagnetic field decays with distance, and when the sensor array is far away from the magnetic source, the signal is weak and the signal-to-noise ratio is low, which poses a challenge for distinguishing the presence of lesions.

When concerning the size of the effective domain, it was found that for cubic sensors, the larger the size, the lower the classification accuracy, which has already demonstrated the negative impact of sensor size on biomagnetic field measurement (Figure 2C). There may be two reasons for this phenomenon: first, under the condition that the distance from the bottom of the sensors to the biomagnetic source is the same, the effective domain of the sensors with a larger height actually includes more area farther from the biomagnetic source, which reduces the signal-to-noise ratio of the measured signal; second, the larger bottom area may cause masking and offsetting effects, which make it more difficult to distinguish the anomalies.

The distance between the center of a cuboid sensor and the main dipole plane is independent of the effective size, so we can distinguish the factors of distance and size more definitely by examining the results of cuboid sensors. In the results shown in the figure, we did not observe a stable reduction in classification accuracy with increasing sensor bottom edge length (Figure 3C), with a fixed height of h=0.5cm.

To further validate the conclusions obtained from comparing the cubic sensor arrays and cuboid sensor arrays, we selected d=4cm, which corresponds to the distance between the ventricular anterior wall and the body surface. Then, we plotted the distribution of classification accuracy in the parameter space of a and h (Figure 4). Based on the graphical results, we can further confirm the aforementioned conclusion, namely that the variation in sensors’ height (h) significantly affects the performance of the sensor array, whereas the variation in sensors’ width (a) does not have a significant impact on the performance of the sensor array. We posit that the potential cause for this outcome may be the mutual cancellation of the effect of sensor array coverage area and the masking and offsetting effects. That is to say in our designed experiment, regardless of whether the sensor has a larger or a smaller bottom edge length, the number of sensors in the sensor array is the same, which further results in the sensor array consisting of larger sensors actually covering a larger area on the body surface. This may have a positive impact on the resolution accuracy, which may offset the negative impact of masking and offsetting effects. This can be further demonstrated by adding channels to the sensor array with smaller sensors so that it covers the same area on the body surface as the sensor array with larger sensors.

Finally, the results mentioned earlier were verified quantitively through simple linear regression (Table 1). For the results given by the sensor arrays with varied d, we selected either the edge length (bottom edge length for cuboid sensor, a) or the distance between the sensor bottom and the magnetic source plane (d) as the regressor, and the classification accuracy as the regressand. For the results given by the sensor arrays with d=4cm and varied a and h, we selected either a or h as the regressor and the classification accuracy as the regressand. To demonstrate the correlation between the performance of the sensor array and the corresponding regressor, we conducted a hypothesis test on the slope parameter. It can be observed that for a sensor array composed of cubic sensors, fixing either a or d, the other variable has a significant effect on the sensor array’s performance (slope parameter is significantly non-zero). For a sensor array composed of cuboid sensors with a fixed h, fixing a, d has a significant effect on the sensor array’s performance. However, the effect of a on the sensor array’s performance is not significant while d is kept invariant. When d is fixed at 4cm, d has a significant effect with a fixed a but a does not have a significant effect with a fixed d. This is consistent with the previous intuitive observations, and the numerical value of the slope parameter itself is also a valuable quantitative indicator that can measure the magnitude of the regressor’s impact on accuracy under corresponding conditions.

**Table 1:** Regression results of accuracy on *a*, *h*, and *d*

Sensor type	Regressor	Control	Slope parameter	Standard error	*t*	*p* value	Significance level
Cubic	*a*	*d* = 2	−0.017	0.000	−17.51	0.000	∗∗∗
		*d* = 2.5	−0.020	0.002	−10.66	0.000	∗∗∗
		*d* = 3	−0.017	0.002	−8.67	0.000	∗∗∗
		*d* = 4	−0.016	0.002	−10.20	0.000	∗∗∗
		*d* = 5	−0.009	0.004	−2.05	0.095	n.s.
	*d*	*a* = 0.5	−0.035	0.002	−18.21	0.000	∗∗∗
		*a* = 1	−0.033	0.003	−10.53	0.002	∗∗
		*a* = 1.5	−0.034	0.003	−12.59	0.001	∗∗
		*a* = 2	−0.024	0.005	−4.67	0.019	∗
		*a* = 2.5	−0.032	0.002	−15.42	0.001	∗∗
		*a* = 3	−0.029	0.002	−16.42	0.000	∗∗∗
		*a* = 4	−0.024	0.005	−5.05	0.015	∗
Cuboid (*h* = 0.5 cm)	*a*	*d* = 2	−0.002	0.002	−0.74	0.492	n.s.
		*d* = 2.5	−0.005	0.005	−0.94	0.389	n.s.
		*d* = 3	−0.004	0.005	−0.79	0.464	n.s.
		*d* = 4	−0.005	0.003	−1.86	0.121	n.s.
		*d* = 5	0.000	0.003	0.09	0.930	n.s.
	*d*	*a* = 0.5	−0.035	0.002	−18.21	0.000	∗∗∗
		*a* = 1	−0.036	0.005	−7.41	0.005	∗∗
		*a* = 1.5	−0.036	0.003	−12.73	0.001	∗∗
		*a* = 2	−0.034	0.006	−6.04	0.009	∗∗
		*a* = 2.5	−0.034	0.005	−6.52	0.007	∗∗
		*a* = 3	−0.031	0.005	−6.27	0.008	∗∗
		*a* = 4	−0.037	0.005	−7.38	0.005	∗∗
Cuboid (*d* = 4 cm)	*a*	*h* = 0.5	−0.005	0.003	−1.86	0.121	n.s.
		*h* = 1	0.003	0.003	1.03	0.350	n.s.
		*h* = 1.5	−0.002	0.003	−0.57	0.596	n.s.
		*h* = 2	−0.000	0.001	−0.08	0.936	n.s.
		*h* = 2.5	−0.001	0.001	−1.11	0.316	n.s.
		*h* = 3	0.001	0.002	0.37	0.728	n.s.
		*h* = 4	0.003	0.004	0.75	0.487	n.s.
	*h*	*a* = 0.5	−0.016	0.001	−18.65	0.000	∗∗∗
		*a* = 1	−0.022	0.002	−10.54	0.000	∗∗∗
		*a* = 1.5	−0.014	0.001	−12.49	0.000	∗∗∗
		*a* = 2	−0.011	0.001	−10.88	0.000	∗∗∗
		*a* = 2.5	−0.010	0.002	−4.24	0.008	∗∗
		*a* = 3	−0.012	0.002	−4.97	0.004	∗∗
		*a* = 4	−0.015	0.004	−4.31	0.008	∗∗

The *t* is calculated through the standard process of t test. The *p* value and significance level are corresponded to two-tailed test, where ∗ shows *p* < 0.05, ∗∗ shows *p* < 0.01, ∗∗∗ shows *p* < 0.001, and n.s. means no significance.

## Discussion

We have established BEST and evaluated the influence of different technical indicators on the performance of biomagnetic field sensors diagnosing small lesions in the tissue. Our results successfully verified the widely accepted assumption that increasing the distance between the sensors and the biomagnetic source has a negative impact on the performance of the sensors. At the same time, our conclusion also demonstrated that, under the condition that the distance between the bottom of the sensors and the magnetic source is the same, making the sensors as “short” as possible has a positive impact on the performance of the sensors. Our results do not directly reflect the influence of the bottom edge length of the sensors on their performance. We speculate that the reason for this phenomenon may be that the negative impact of masking and offsetting effects is offset by the positive impact of the sensor array coverage area. These findings could give some elicitation for the development of biomagnetic field sensors as well as some guidance on the choice of sensors while constructing measuring instruments.

Furthermore, BEST is a versatile framework for evaluating different sensors quantitatively, which means complicated technical indicators of the sensor can be set and studied in BEST, and the ranking of accuracy always has physical significance. This could facilitate both the sensor’s developers and the sensor’s consumers making their choice. They could simply enter the sensor’s indicators they have to choose among and take the ranking of accuracy for reference.

Based on BEST, more complicated applications can be developed. For the field source, we only construct an abstract static model in this study. Nowadays, dynamic models for bioelectrophysiological activities have been developed, and different lesions have been simulated in these models.^23^^,^^27^ While incorporating these more specific models, biomagnetic field source models and magnetic field forward modeling algorithm, BEST can be easily adapted to more complex application scenarios. While dealing with time series of biomagnetic signals generated by dynamics models, time-frequency analysis including short-time Fourier transform and wavelet transform can be applied to the time series and the network immediately following does not even have to be substantially reformed while only substituting the original inputs with time-frequency matrices. For the classifier, CNNs with different network structure and even classifiers based on completely different algorithm can be applied and evaluated. In this paper, we applied DenseNet as the main model. For the biomagnetic field sensors, indicators that can be studied in this framework are not limited to those studied in this paper. Deep insights into the principle of detecting magnetic field and the source of noise can be incorporated into this framework. For example, temporal and spatial characteristics of sensor’s sensitivity can be incorporated by a coefficient ρ(r;t).

We believe that in the near future, with more accurate models of the biomagnetic field and the sensor, and more data, we have the potential to establish a complete system of performance evaluation indicators for the sensor through statistical methods including regression based on BEST. With the set of indicators, we can obtain a reasonable estimate of the sensor’s performance in various aspects. Research in the fields proposed above will further guide researchers to choose their technology roadmap while developing magnetometers and biomagnetic devices.

### Limitations of the study

In this study, the simulation of MCG was purely phenomenological. Although the simulated method and results could correspond to the real situation to some extent, they might lack persuasiveness in certain aspects. Our sensor model also did not fully correspond to a specific type of sensors. The sensitivity of a certain type of sensors may be quantitively related to their effective size, an effect that was not considered in our study. We employed a CNN (Figure S2) to provide quantitative metrics. While CNNs are commonly used for the classification of biological magnetic signals and can simulate diagnostic research effectively, their results can be unstable and computationally demanding, which may pose challenges to the reproducibility of experimental results. In particular, the verification accuracy of CNNs may not accurately reflect the features in the data due to overfitting.

## STAR★Methods

### Key resources table

**Table:** 

REAGENT or RESOURCE	SOURCE	IDENTIFIER
**Deposited data**		

Random noises and dipole sets	This paper	Mendeley Data: https://doi.org/10.17632/6tv48c4tvr.1
Classifying results	This paper	Mendeley Data: https://doi.org/10.17632/6tv48c4tvr.1

**Software and algorithms**		

Anaconda	Anaconda	https://www.anaconda.com/
Jupyter Notebook	Jupyter Hub	https://jupyter.org/
Thonny	University of Tartu	https://thonny.org/
Python	Python Software Foundation	https://www.python.org/
Tensorflow	TensorFlow	https://www.tensorflow.org/
Algorithm for simulated testing	This paper	Mendeley Data: https://doi.org/10.17632/6tv48c4tvr.1
Algorithm for data processing	This paper	Mendeley Data: https://doi.org/10.17632/6tv48c4tvr.1

**Other**		

Weiming-1 cluster	High Performance Computing Platform of Peking University	https://scow.pku.edu.cn/

### Resource availability

#### Lead contact

Further information and requests for resources should be directed to and will be fulfilled by the lead contact, Hong Guo (hongguo@pku.edu.cn).

#### Materials availability

This paper did not generate new unique materials.

#### Data and code availability

• All original data have been deposited at Mendeley Data and are publicly available as of the date of publication. The DOI is listed in the key resources table.
• All original code has been deposited at Mendeley Data and is publicly available as of the date of publication. The DOI is listed in the key resources table.
• Any additional information required to reanalyze the data reported in this paper is available from the lead contact upon request.

### Method details

#### Experimental design

We used a fixed main dipole to simulate the biomagnetic field generated by the normal electrophysiological activities and some randomly distributed small dipoles to simulate the biomagnetic field generated by defects in the tissue. Measuring the main dipole gave the normal signals and measuring the main dipole and small dipoles simultaneously gave the abnormal signals. The magnetic field at a particular point in space was computed individually, and the sensor measurement was obtained by averaging the z-component of the magnetic field over all field points within the sensor's effective domain, with an added value that simulated the noise. A total of 10,000 sets of small dipoles and noises was generated and the measurement was repeated 10,000 times accordingly, giving the normal data set and abnormal data set. The first 8,000 data in both sets was utilized as the training data set and the rest as the testing data set without cross validation. We trained a CNN to obtain a classifier to tell whether a signal was from the normal data set or abnormal date set. The model was iterated for 30 times, and we selected the one that achieves the best classification performance on the validation set and recorded its accuracy.

#### Current dipole model

Current dipole model is a wildly applied model as the volume source in simulating biomagnetic signals. During the excitation propagation in the tissue, excitable cells are depolarized and repolarized in a relatively fixed order and membrane potential gradient arises between the depolarized cells and unexcited cells and thus generates current dipoles with the depolarized area as current source and the unexcited area as current sink. The magnetic field due to a current dipole $\left(\rho \right)$ at the field point $\left(r_{f}\right)$ is calculated from

$$B\left(r_{f}\right)=\mu_{0}\frac{\rho \times r}{4\pi r^{3}}\text{,}$$

where $\mu_{0}$ is the permeability of free space, r is the displacement vector from ρ to $r_{f}$ with a magnitude r, and × represents the cross product.

In this study, we used a main dipole with high current density to simulate the magnetic field generated by normal tissue and different amounts of dipoles with low current density distributed randomly besides the main dipole as the representation of different defects in the tissue. When attempting to simulate the measurement results, we defined the sensor’s positions and effective areas, and uniformly selected field points within the effective area. Subsequently, we will provide a specific account of this procedure and the coordinate system employed is consistent with the definition provided in the preceding text.

First, we defined the coordinates of the field points’ projections onto the x−y plane. The rule was to take (0cm,0cm), (24cm,0cm), (24cm,24cm), (0cm,24cm) as start points, take 1mm as step size, and uniformly select points within the rectangle defined by the four points above.

Next, we defined the position of the sensors in the 6×6 senor array. The bottoms of the sensors’ effective areas are in the same plane parallel to the x−y plane, whose projections onto the x−y plane coincide with (2cm,2cm), (2cm,6cm), (2cm,10cm), (2cm,14cm), (2cm,18cm), (2cm,22cm), (6cm,2cm), (6cm,6cm), (6cm,10cm), (6cm,14cm), (6cm,18cm), (6cm,22cm), (10cm,2cm), (10cm,6cm), (10cm,10cm), (10cm,14cm), (10cm,18cm), (10cm,22cm), (14cm,2cm), (14cm,6cm), (14cm,10cm), (14cm,14cm), (14cm,18cm), (14cm,22cm), (18cm,2cm), (18cm,6cm), (18cm,10cm), (18cm,14cm), (18cm,18cm), (18cm,22cm), (22cm,2cm), (22cm,6cm), (22cm,10cm), (22cm,14cm), (22cm,18cm), (22cm,22cm). By combining these projected points with the bottom edge lengths of the effective areas of the sensors, we initially determined the projection positions on the x−y plane of all the field points that were to be included in the computation.

Finally, we augmented the projected coordinates of all the field points with a third dimension by taking into account the distance between the plane of the sensors’ bottoms and the torso, d, as well as the height of the sensors, h, thereby obtaining the coordinates of all the field points. The vertical distance between adjacent field points in the z-direction is also 1mm. For example, for a d=2cm sensor array consisting of h=0.5cm sensors, the corresponding z-coordinates added were 2.0cm, 2.1cm, 2.2cm, 2.3cm, and 2.4cm.

Consequently, we simulated different sensor arrays based on the three parameters of d, a and h. Within the effective area of each sensor, we selected a field point every 1mm and calculated the mean field strength at these selected points to simulate the sensor’s measurements. The field point selection was sufficiently dense that even within the effective area of the smallest 0.5cm×0.5cm×0.5cm sensor, 125 field points were available.

#### Biomagnetic field sensors

To evaluate the size effect on the performance of biomagnetic field sensors, we simulated sensors with different effective detection areas rather than using single field points to represent sensors. In concrete, we chose a series of field points distributed uniformly within a particular area representing the effective detection area of the sensor and the signal detected by the sensor was simulated by averaging the magnetic field generated by the dipole model at all field points (32), i.e.

$$B_{i}=\frac{\sum_{k=1}^{n}B_{ki}}{n}\left(i=x,y,z\right)\text{,}$$

where k is the field point number and n is the total number of field points.

#### CNN test

CNN acts as an effective tool in the classification of biomagnetic signals. After CNN is trained by the training set, the classification accuracy of the validation set relies on not only the performance of the network but also the significance of features for classification in the dataset. When CNN is applied to signals generated by the same sources but measured with different devices, the accuracy of classification by the same CNN could act as an index to evaluate the quality of the measured signals and, in other words, the performance of measuring devices. In this line of thought, we applied CNN to weigh the influence of different technical indicators on the measuring efficiency of the biomagnetic field sensors.

We applied a self-designed CNN based on Pytorch which has been validated in our previous work. The training was primarily conducted on the Weiming-1 cluster, which is housed at Peking University's High-Performance Computing Center.

The CNN consists of several components (Figure S2). Firstly, the input layer extracts feature from the input data using a 3×3 convolutional layer and normalizes the output using BatchNorm and applies the ReLU activation function for non-linearity. Secondly, an SELayer is added after the input layer to adjust the input feature maps with channel-wise attention mechanism to improve the model's performance and robustness. Thirdly, several dense blocks are included, where each dense block contains multiple dense layers, which are the basic units of this model comprising of two convolutional layers and an SELayer, effectively extracting and reusing feature information. Fourthly, a transition layer is added between each dense block to reduce the number of output channels by half and perform downsampling using average pooling operation to reduce computation and memory usage. Finally, the feature map is transformed into a one-dimensional vector using global average pooling and fed into a fully connected layer to output the final classification result.

In our study, the model was trained for 30 epochs on data simulated from different sensor arrays. The training was conducted on the Weiming-1 cluster housed at High Performance Computing Platform of Peking University. In the case of (d=2cm,a=0.5cm,h=0.5cm), (d=5cm,a=4cm,h=4cm) and (d=5cm,a=4cm,h=0.5cm), we reperformed the training separately with the corresponding datasets and recorded the average loss of the training set and validation set at the end of each epoch during the training process. Regarding the specific form of the loss function, we employed the cross-entropy loss function used during the training process, which is defined as

$$L=\frac{1}{N}\sum_{i}L_{i}=\frac{1}{N}\sum_{i}-\left[y_{i}\cdot \log\left(p_{i}\right)+\left(1-y_{i}\right)\cdot \log\left(1-p_{i}\right)\right]\text{,}$$

where N is the quantity of samples, N=16,000 for the training set, N=4,000 for the validation set, $y_{i}$ is the label of sample i, $y_{i}=0$ for measurements of normal signals, $y_{i}=1$ for measurements of abnormal signals, $p_{i}$ is the possibility of predicting sample i to be a measurement of the normal signal. Based on the experimental results, it can be observed that for these three relatively extreme cases, the model successfully converged after 30 training epochs without overfitting (Figure S3).

### Quantification and statistical analysis

The CNN provided classification results for the validation set using a confusion matrix (Figure S3A). Thirty classification models were trained through 30 rounds of training for each dataset, corresponding to 30 confusion matrices. The classification accuracy (A) corresponding to each confusion matrix was calculated through

$$A=\frac{TP+TN}{TP+FP+FN+TN}\text{.}$$

After obtaining the highest classification accuracy for each dataset, we processed the results with two-dimensional interpolation for visualization. In the two-dimensional interpolation, the distance between the sensor bottoms and the magnetic source plane (d), is used as the x-axis coordinate, with a range of [2cm,5cm] divided into 300 intervals. The edge length (bottom edge length for cuboid sensor, a), which is used as the y-axis coordinate, has a range of [0cm,4cm] and is divided into 400 intervals. For the results of cubic sensors, we used cubic interpolation. However, for the results of cuboid sensors, cubic interpolation caused severe distortion at the edges of the resulting images, so linear interpolation was used instead.

To present the results more clearly, we controlled one variable in a and d, plotted a scatter plot of accuracy against the other variable, and performed regression analysis. Since we had no prior knowledge of the relationship between accuracy and a or d, we chose the simple linear mode $y=\beta_{0}+\beta_{1}x$ as the regression model, with a or d as the regressor and accuracy as the regressand. To further describe the correlation between accuracy and a or d, we conducted a t-test on the slope parameter. The null hypothesis of the hypothesis test was $H_{0}:\beta_{1}=0$, and the alternative hypothesis was $H_{1}:\beta_{1}\neq 0$. Regression and hypothesis testing were performed using Stata, and the output results of Stata were summarized in Table 1.
