# Supplementary material for: Optimizing biomagnetic sensor performance through in silico diagnostics: A novel approach with BEST (Biomagnetism Evaluation via Simulated Testing)
Source: iScience. 2024 Jun 4;27(7):110167. doi: 10.1016/j.isci.2024.110167 (PMC11226959; doi:10.1016/j.isci.2024.110167)
Supplement: Document S1. Figures S1–S3 [file mmc1.pdf]

iScience, Volume *27*

Supplemental information

Optimizing biomagnetic sensor performance through *in silico* diagnostics: A novel approach with BEST (Biomagnetism Evaluation via Simulated Testing)

Chenxi Sun, Yike Liang, Xiao Yang, Biying Zhao, Pengju Zhang, Sirui Liu, Dongyi Yang, Teng Wu, Jianwei Zhang, and Hong Guo

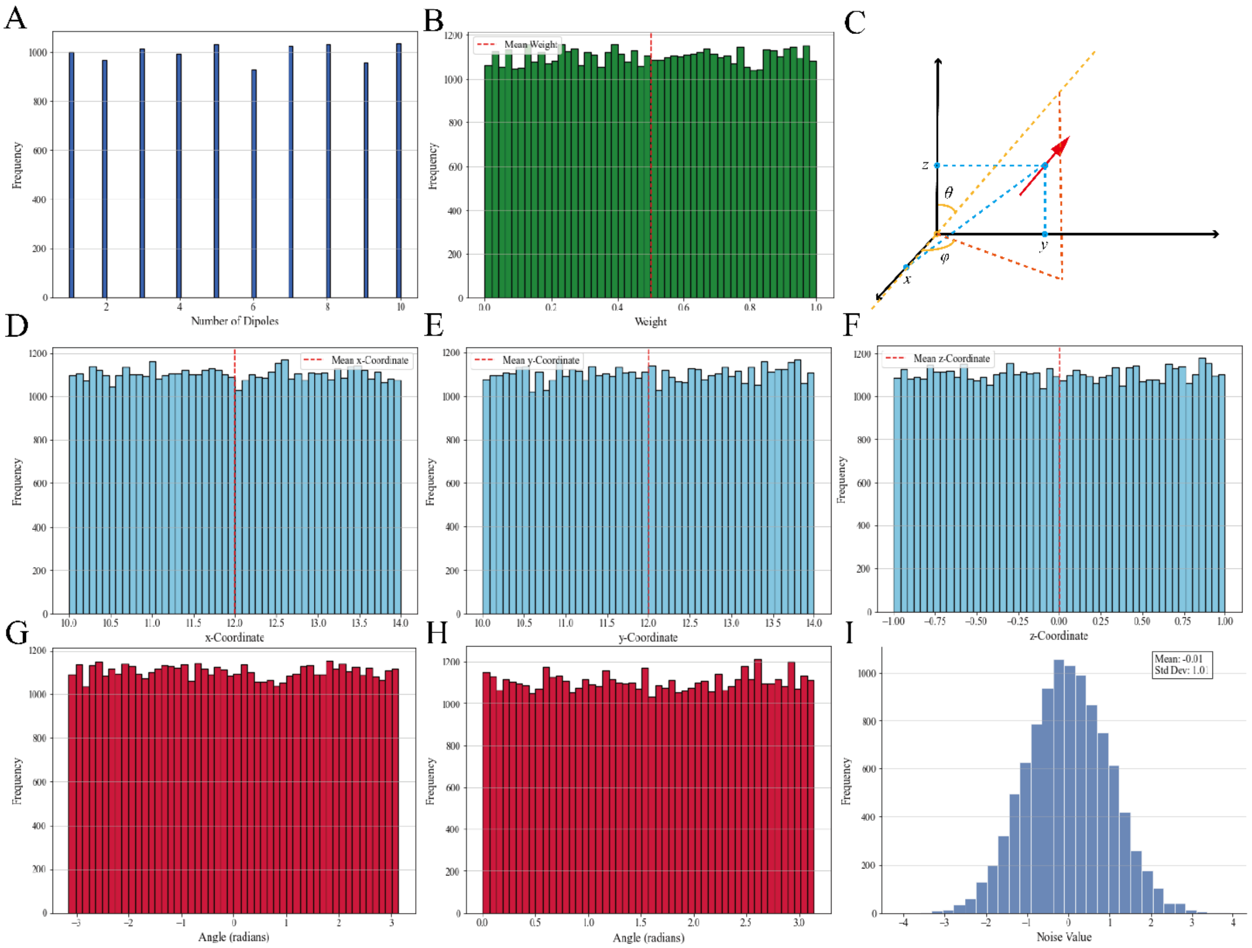

Figure S1. The statistical properties of the added random dipoles and noises, related to STAR Methods. (A) Distribution of the number of random dipoles in each model of a defective heart. (B) Distribution of the weight of random dipoles, where the red dash line shows the mean weight. (C) Definitions of main parameters describing the characteristics of random dipoles, including x-, y-, and z-coordinates, as well as θ and φ. (D)-(F) Distribution of the x-, y-, and z-coordinates, where the red dash lines show the mean coordinates. (G)-(H) Distribution of θ and φ. (I) Distribution of the random noises.

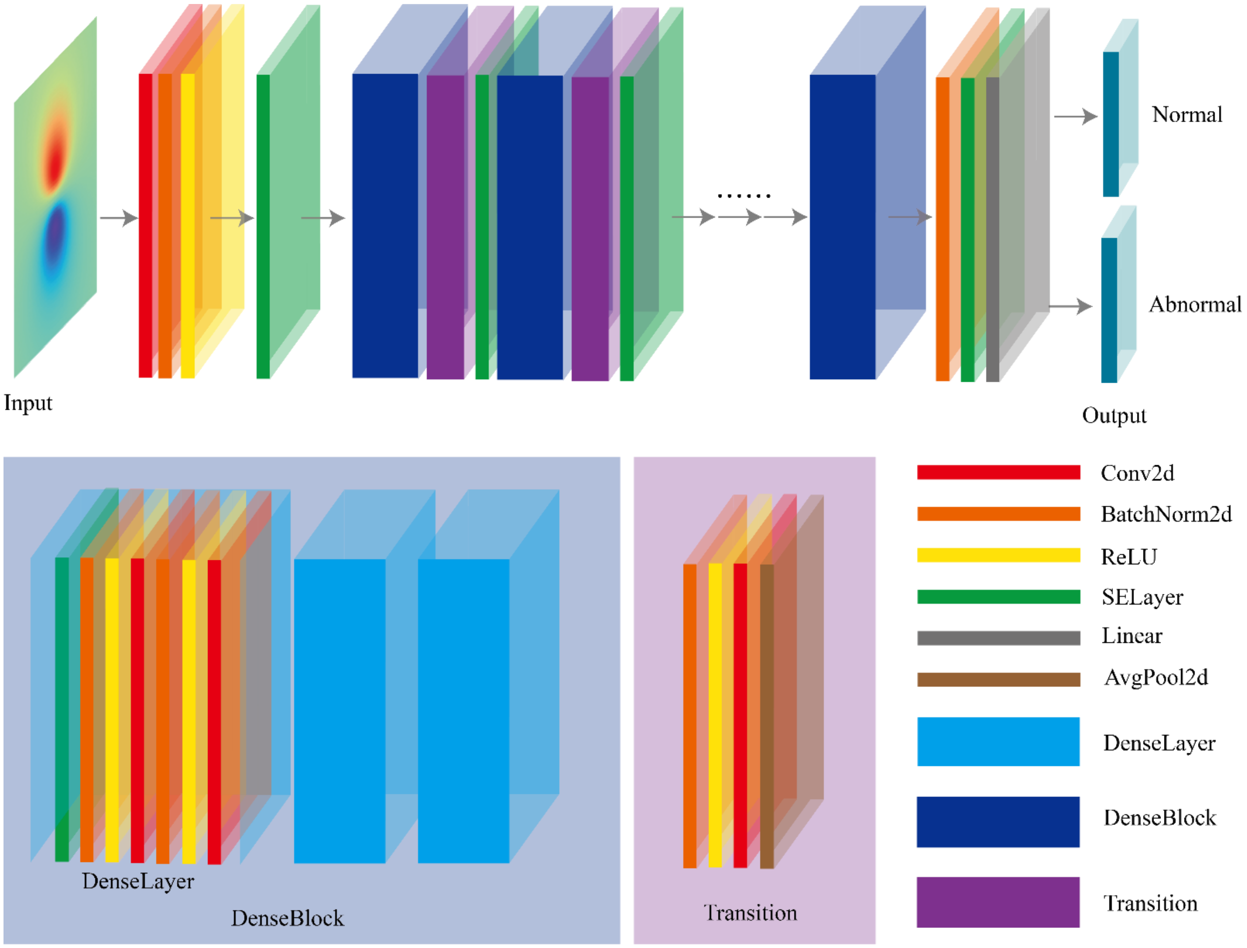

Figure S2. Visualization of the CNN architecture applied in this study. The network was constructed using the Pytorch framework, related to STAR Methods. The upper portion of the image illustrates the overall structure of the network, with the DenseBlock and Transition components consisting of multiple layers, which are thoroughly depicted in the lower part of the image.

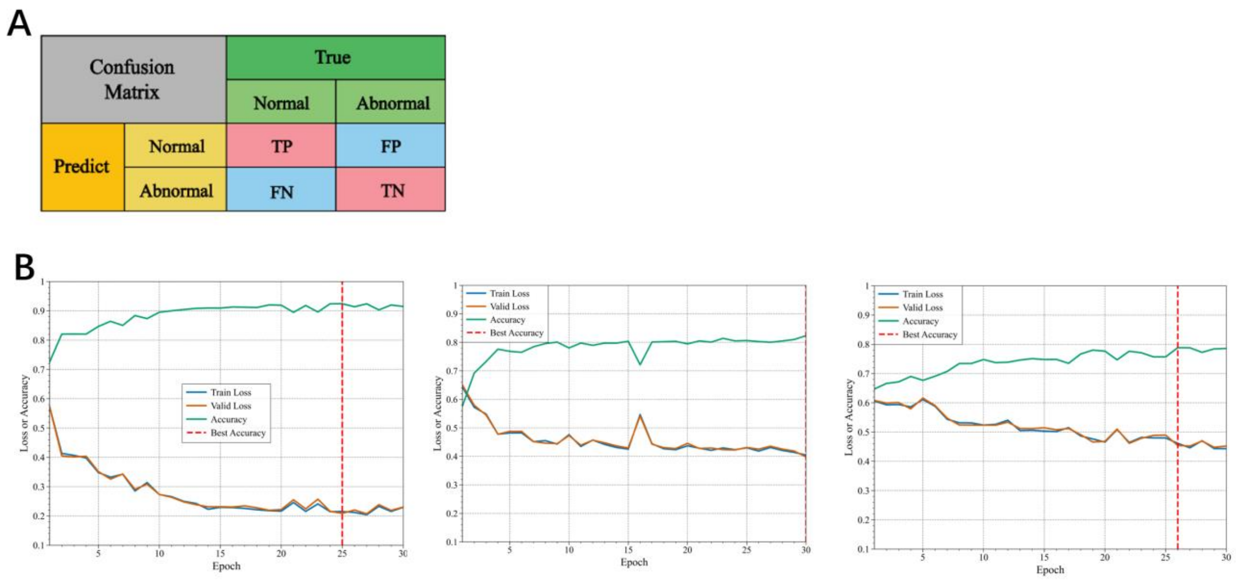

Figure S3. Quantitative description of the training results, related to STAR Methods. (A) Definition of the data in the confusion matrix of the model output. (B) The variations of training loss, validation loss, and accuracy with epochs during the model training process. From left to right, the figures correspond to $(d = 2\ \mathrm{cm}, a = 0.5\ \mathrm{cm}, h = 0.5\ \mathrm{cm})$, $(d = 5\ \mathrm{cm}, a = 4\ \mathrm{cm}, h = 0.5\ \mathrm{cm})$, and $(d = 5\ \mathrm{cm}, a = 0.5\ \mathrm{cm}, h = 0.5\ \mathrm{cm})$ cases, respectively. The red dash line shows the epoch achieving the best accuracy. For each case, both the training loss and validation loss converged to a stable level before 30 epochs, and the validation loss did not significantly increase.
